# Gut microbiota contributes to lignocellulose deconstruction and nitrogen fixation of the larva of *Apriona swainsoni*

**DOI:** 10.3389/fphys.2022.1072893

**Published:** 2022-12-23

**Authors:** Lei Zhang, Tian Zhuang, Mengxue Hu, Shuwen Liu, Daqiang Wu, Baozhong Ji

**Affiliations:** ^1^ Co-Innovation Center for the Sustainable Forestry in Southern China, College of Forestry, Nanjing Forestry University, Nanjing, China; ^2^ Department of Pathogenic Biology and Immunology, College of Integrated Chinese and Western Medicine, Anhui University of Chinese Medicine, Hefei, China; ^3^ The Administration Bureau of Dr. Sun Yat-sen’s Mausoleum, Nanjing, China

**Keywords:** Apriona swainsoni, larvae, gut segments, gut microbiota, lignocellulose deconstruction, nitrogen fixation

## Abstract

*Apriona swainsoni* is a vital forest pest prevalent in China. The larvae of *A. swainsoni* live solely in the branches of trees and rely entirely on the xylem for nutrition. However, there is still a lack of in-depth research on the gut microbiota’s use of almost nitrogen-free wood components to provide bio-organic macromolecular components needed for their growth. Thus, in this study, the metagenome, metaproteome, and metabolome of the *A. swainsoni* larvae in four gut segments (foregut; midgut; anterior hindgut; posterior hindgut) were analyzed by the multi-omics combined technology, to explore the metabolic utilization mechanism of the corresponding gut microbiota of *A. swainsoni*. Firstly, we found that the metagenome of different gut segments was not significantly different in general, but there were different combinations of dominant bacteria and genes in different gut segments, and the metaproteome and metabolome of four gut segments were significantly different in general. Secondly, the multi-omics results showed that there were significant gradient differences in the contents of cellulose and hemicellulose in different segments of *A. swainsoni*, and the expression of corresponding metabolic proteins was the highest in the midgut, suggesting the metabolic characteristics of these lignocellulose components in *A. swainsoni* gut segments. Finally, we found that the C/N ratio of woody food was significantly lower than that of frass, and metagenomic results showed that nitrogen fixation genes mainly existed in the foregut and two hindgut segments. The expression of the key nitrogen fixing gene *nifH* occurred in two hindgut parts, indicating the feature of nitrogen fixation of *A. swainsoni*. In conclusion, our results provide direct evidence that the larvae of *A. swainsoni* can adapt to the relatively harsh niche conditions through the highly organized gut microbiome in four gut segments, and may play a major role in their growth.

## 1 Introduction

After millions of years of evolution thrive of numerous insects including forest pests is closely tied to gut microbiota associations with hosts (Schmidt et al., 2021). The possible theory of living niche adaptation and competition for phytophagous insects with host plants might help in understanding interspecies relationships and species-specific nutrient requirements of insects (Shapira, 2016; Ceja-Navarro et al., 2019). During invasive events of phytophagous pests into the new niches, competitions are usually intense due to nutrient stress and space limitation, and the pressure of plant host and niche selection (Bird et al., 2019). Only the pests with the better niche occupancy capability can obtain the dominant community in the plant host (Warnecke et al., 2007). More than the genome-level adaptive coevolution, the gut microbiota may also make advantage of phytophagous pests utilizing the host plant (Munoz-Benavent et al., 2021). The gut microbiota of the insect kingdom coevolved with the plants that insects live in.

Hammer and Bowe claimed the “gut microbial facilitation hypothesis” explained that the adaptability of phytophagous pests to parasitic plants is related to the gut microbiota variation (Hammer and Bowers, 2015; Girard et al., 2022). Over millions of years of evolution, insects and gut microbes have coevolved, interacted, and cooperated into an inseparable symbiotic correlation (Hammer and Bowers, 2015). Intestinal microbe metabolic activity and community structure of insects are adapted to and influenced by the host-gut microenvironment (Brandon et al., 2021). Various gut microorganisms may help insects metabolize the nutrients synthesized by host plants (Bird et al., 2019; Mason, 2020; Zhang Q. C et al., 2022). However, the gut microbiota is also found to be a burden in the leaf beetle’s larval development of *Plagiodera versicolora* (Ma et al., 2021). Previously, (Ceja-Navarro et al., 2019) demonstrated that gut anatomical properties and microbial functional assembly of a wood-feeding beetle *Odontotaenius disjunctus* enable lignocellulose deconstruction and colony subsistence on a vitally nutrient-poor woody diet (Ceja-Navarro et al., 2019). However, related research on Cerambycid species, the other important forest pest, remains elusive.

Apriona swainsoni (Coleoptera: Cerambycidae) is a phytophagous pest against forests and an important threat to the trees of *Sophora* japonica that is the afforestation tree species in China (Wei et al., 2013; Munoz-Benavent et al., 2021; Zhang et al., 2022b). *A. swainsoni* also is the main threat of *Caesalpinia decapetala* which is a source of Traditional Chinese Medicine (Zengin et al., 2021). The adult and larvae of *A. swainsoni* attack healthy trees and spend most of their life as larvae living inside main branches and tree trunks, usually causing high mortality in the host plant (Zhang S. K et al., 2022). In several provinces of China, *A. swainsoni* is listed as a quarantine forest pest (Wei et al., 2013). Using 16S rDNA sequencing, Zhang and colleagues found that the gut microbiome in the whole gut tended to play a part in host niche competition of *A. swainsoni* and *A*priona *germari* (Zhang Q. C et al., 2022)*.* Same as *O. disjunctu,* the whole gut of *A. swainsoni* consists of four gut segments i.e., foregut, midgut, anterior hindgut, and posterior hindgut. However, the relationship between gut microbiota in four gut segments of the digestive tract and the nutrition absorption mode of *A. swainsoni* is still lacking.

The larvae of *A. swainsoni* live in woody branches and pupate for nearly 2 years, and finally emerge as adults. In the larval stage, the insect only takes the transported woody components as its food source, and nutritional components are only cellulose, hemicellulose, and lignin in the xylem, which is extremely simple and hard to digest for the animal kingdom. The larvae live in the xylem of trees, which is mostly composed of lignocellulose and has quite low nitrogen content (Park et al., 2007; Nagamine et al., 2016). As a relatively large insect, the longicorn larvae require a large amount of organic nitrogen to synthesize proteins and nucleotides for their living. Thus, the organic nitrogen of *A. swainsoni* larvae only can come from the nitrogen fixation performed by its gut microbiota. Therefore, the relationship between gut microbiota and lignocellulose digestion and nitrogen fixation of *A. swainsoni* larvae is worth investigating.

Here, we have investigated how spatially separated gut microbiota functionality facilitates the deconstruction and transformation of the main nutrient, lignocellulose, in the four gut segments of the digestive tract of *A. swainsoni* larvae. Using metagenome and metaproteome analyses, we analyzed the relation of metagenomic composition and functional metaproteome with lignocellulose digestion and nitrogen fixation, and the distribution of vital microbial genes and proteins throughout the digestive gut segment of *A. swainsoni*. Metabolomics results of the gut segments supported the production of metabolites predicted by metagenomic and metaproteomic of lignocellulose digestion. Therefore, our findings suggest that the *A. swainsoni* larvae have evolved a featured and well-organized digestive tract with specialized gut segments for nitrogen fixation and lignocellulose deconstruction.

## 2 Materials and methods

### 2.1 Sampling collection and anatomy

*Apriona swainsoni* is a pest widely distributed in southeast China, which is harmful to the economical and natural trees (Zhang S. K et al., 2022). Totally 150 fourth or fifth-instar larvae of *A. swainsoni*, which are in the middle stage of larva development with typical physiology phenotype and metabolism status, were collected in the *C. decapetala* wood branches. The insect species were recognized by the specified head and pronotum of the larva of *A. swainsoni*, and four gut segments of all the collected larvae were dissected for further examination. Each gut segment for multi-omics analyses contains four samples of biological repeats. Every gut segment sample consists of 8–12 dissected gut segment organs to meet the quantity requirement of multi-omics analyses. Then the *C. decapetala* branches were used for validating C/N ratio of larvae food. The gut segments with lumen contents and plant branches were stored at −80°C for subsequent analysis.

### 2.2 DNA isolation, library construction, and metagenome sequencing

QIAamp^®^ Fast DNA Stool Mini Kit (Qiagen, Hilden, Germany) was used to isolate total DNA of samples following the manufacturer’s instructions. A NanoDrop2000 spectrophotometer (Thermo Fisher Scientific, Waltham, MA, United States) and agarose gel electrophoresis were used to assess DNA concentration and integrity. The concentration of extracted DNA was 20–68 ng/μl. Agencourt AMPure XP beads (Beckman Coulter Co., United States) were used to clean up the DNA fragmented with S220 Focused-ultrasonicators (Covaris, United States). Afterward, libraries were constructed using the TruSeq Nano DNA LT Sample Preparation Kit (Illumina, San Diego, CA, United States). Metagenome sequencing and analysis were performed by OE Biotech Co., Ltd. (Shanghai, China).

### 2.3 Bioinformatic analysis

Trimmomatic was used to trim and filter FastQ sequences (Bolger et al., 2014). Host pollution control was performed due to the DNA was extracted from a host-related sample. To align the post-filtered pair-end reads with the host genome, BWA was used (Li and Durbin, 2010) and the aligned reads were discarded. After valid reads were obtained, the genome was assembled using SOAPdenovo2 (Luo et al., 2012). Use gaps inside the scaffold as breakpoints to interrupt the scaffold into new contigs (Scaftig), and these new Scaftigs with lengths > 200 bp (or 500 bp) were retained. ORF prediction of assembled scaffolds using prodigal was performed and translated into amino acid sequences. The non-redundant gene sets were built for all predicted genes using CDHIT (Fu et al., 2012). The clustering parameters were 95% identity and 90% coverage. The longest gene was selected as the representative sequence of each gene set. Clean reads of each sample were aligned against the non-redundant gene set (95% identity) using Bowtie2 (Langmead and Salzberg, 2012), and the abundant information of the gene in the corresponding sample was counted. Then, the gene set representative sequence (amino acid sequence) was annotated with NR, KEGG, COG, SWISSPROT, and GO database with an e-value of 1e-5. Taxonomy for the species was obtained from the NR Library’s corresponding taxonomy database, and the abundance of the species was calculated using the corresponding abundance of the genes. At each taxonomy level of Domain, Kingdom, Phylum, Class, Order, Family, Genus, and Species., abundance statistics were performed to construct the abundance profile.

The gene sets were compared with the CAZy database (Drula et al., 2022) using the corresponding tool hmmscan to obtain information on the carbohydrate-active enzyme corresponding to the gene. R software (v3.2.0) was used for the PCA analysis and plotting of species or functional abundance spectra.

For bin analysis, Megahit (Li et al., 2015) was used to assemble clean sequencing data and remove hosts to obtain contigs. Bowtie2 (Langmead and Salzberg, 2012) and Samtools (Li et al., 2009) were respectively used for comparison and format conversion. After obtaining the depth data of Contigs, Contigs above 1,500 bp were used for bin classification and Metawrap (Uritskiy et al., 2018) software was used. Three built-in software algorithms, Maxbin2 (Wu et al., 2016), MetaBAT2 (Kang et al., 2019), and Concoct (Lu et al., 2017), are used to perform macro factor component bins, and the best results are obtained from highly similar bins, and non-redundant bins are output. The Lineage_WF function in ChecKM software (Parks et al., 2015) was applied to evaluate the quality of the box sorting results, and to obtain the completion degree, pollution degree, species classification, and other information of each box. Bins with a screening completion degree greater than 50% and a contamination degree less than 10% were used for subsequent analysis. Based on NCBI_tax and NCBI_nt databases, Taxator-TK (Droge et al., 2015) was used to annotate each contig species and then estimate the overall bin species.

### 2.4 Sample preparation for metaproteomic and metabolomics

The contents of the intestinal cavity of larvae from the four main regions of the digestive tract were homogenized and centrifuged at 21,000 g for 10 min at 4°C. Then the supernatant was transferred to methanol/chloroform aqueous solution, and was remove the upper water-soluble phase which was put into a labeled glass bottle with completely dry for metabolomics analysis. Then, the settled protein was added to 1 ml of ice-cold methanol. The sample was centrifuged again at 10,000 g for 10 min to precipitate the protein group, the methanol was removed, and the sample was placed in the fume hood to dry. Sample lysate was added to the precipitation, and the protease inhibitor PMSF was added to make the final concentration of 1 mM. The solution was centrifuged twice at 12,000 g at 4°C for 10 min to obtain the supernatant. The supernatant is the total protein solution, which shall be determined for protein concentration and stored at −80°C for later use. The concentration of extract protein was 1.2–8.2 μg/μl.

### 2.5 Metaproteome identification and bioinformatics analysis

A Q-Exactive HF mass spectrometer (Thermo, United States) equipped with a Nanospray Flex source (Thermo, United States) was used to perform the metaproteome identification. Samples were loaded and separated by a C18 column (15 cm × 75 µm) on an EASY-nLCTM 1200 system (Thermo, United States). The flow rate was 300 nl/min and the linear gradient was 75 min (0–63 min, 5%–45% B; 63–65 min, 45%–90% B; 65–75 min, 90% B; mobile phase A = .1% FA in water and B = .1% FA in ACN). The AGC target value was set at 3e6, and full MS scans were acquired between 350 and 1,500 m/z with a mass resolution of 60,000. With a collision energy of 32, the 20 most intense peaks in MS were fragmented by higher-energy collisional dissociation (HCD). MS/MS spectra were obtained with a resolution of 45,000 with an AGC target of 2e5 and a max injection time of 80 ms. The Q Exactive HF dynamic exclusion was set for 30.0 s and run under positive mode. The raw data was thoroughly searched against the sample protein database using ProteomeDiscoverer (v.2.4). The database was searched with the specificity of Trypsin digestion. In the database search, alkylation on cysteine was considered a fixed modification. For the protein quantification method, the TMT labeling method was selected. The false discovery rate (FDR) was set at .01 globally, and at least two peptides were required for each protein group to be quantified.

### 2.6 Metabolomics identification and bioinformatics analysis

An accurately weighed sample of 60 mg was transferred to a 1.5 ml Eppendorf tube. Two small steel balls were added to the tube. For each sample, an internal standard (2-chloro-l-phenylalanine in methanol, .3 mg/ml) and an extraction solvent (4/1, v/v) were added. Samples were stored at −80°C for 2 min and then grinded at 60 Hz for 2 min, ultrasonicated at ambient temperature (25°C–28°C) for 10 min, and stored at −20°C for 30 min. The extract was centrifuged at 15,000 g, 4°C for 15 min. With the aid of a freeze concentration centrifugal dryer, 300 μl of supernatant was dried in a brown and glass vial. 400 μl mixture of methanol and water (1/4, vol/vol) was added to each sample. Then the samples were vortexed for 30 s, then placed at 4°C for 2 min. Samples were centrifuged at 15,000 g, 4°C for 5 min. The supernatants from each tube were collected using crystal syringes, filtered through .22 μm microfilters, and transferred to LC vials. The vials were stored at −80°C until LC-MS analysis. QC samples were prepared by mixing aliquots of all samples to be a pooled sample. The analytical instrument for this experiment is a liquid mass coupled system composed of Dionex U3000 UHPLC ultra-performance liquid tandem QE Plus high-resolution mass spectrometer.

Progenesis QI software (Waters Corporation, Milford, United States) was used to analyze the acquired LC-MS raw data with the following parameters. The precursor tolerance was set to 5 ppm, fragment tolerance was set to 10 ppm, and retention time (RT) tolerance was set to .02 min. Internal standard detection parameters were deselected for peak RT alignment, isotopic peaks were excluded for analysis, the noise elimination level was set at 10.00, and the minimum intensity was set to 15% of base peak intensity. The Excel file was obtained with three-dimension data sets including m/z, peak RT and peak intensities, and RT–m/z pairs were used as the identifier for each ion. The resulting matrix was further reduced by removing any peaks with a missing value (ion intensity = 0) in more than 50% of samples. Data QC (reproducibility) was performed by the internal standard. Based on public databases such as http://www.hmdb.ca/; http://www.lipidmaps.org/ and self-built databases progenesis QI software was used to identify the metabolites. The differential metabolites among groups were determined based on the combination of a statistically significant threshold of *p* values from a two-tailed Student’s *t*-test on the normalized peak areas and variable influence on projection (VIP) values, where metabolites with and *p* values less than .05 and VIP values larger than 1.0 were considered as differential metabolites.

### 2.7 Carbon and nitrogen concentrations in wood and gut tissue

Frass samples obtained from five larvae and wood samples were loaded in 8 mm × 5 mm tin capsules using 10–20 mg for nitrogen quantification and 2 mg for carbon quantification. A zero blank autosampler connected to an ECS 4010 Element Analyzer (Costech Analytical) coupled to a Delta V Plus isotope ratio mass spectrometer (Thermo Fisher Scientific) was used to identify the carbon and nitrogen concentration of the samples. The analytical precisions on concentration yields were 70.14 ± 1.29 wt% (1 s; *n* = 7) for carbon and 4.83 ± .09 wt% (1 s; *n* = 10) for nitrogen based on the laboratory atropine standard sample.

### 2.8 Hematoxylin-eosin staining

The four gut region samples of larvae were isolated and dissected in a sterile environment, and the guts were immediately flushed with 4% paraformaldehyde. After flushing, the samples were fixed in 5 times the volume of 4% paraformaldehyde buffer for at least 72 h. After dehydration and paraffin embedding, 5 mm gut tissue sections were taken for Hematoxylin-eosin (H & E) staining. The morphology of gut tissue was observed under an upright microscope (OLYMPUS BX51, Tokyo, Japan).

### 2.9 RT-PCR

The gut tissues from each gut region were isolated in a sterile environment. Total RNA was extracted from gut tissue using a Trizol reagent (Ambion, United States). The concentration of extracted total RNA was 35–80 ng/μl. FastQuant RT Kit reverse transcription kit (TIANGEN, Beijing, China) was applied to reverse RNA into complementary DNA (cDNA). *NifH* and *ureC* genes were amplified by RT-PCR using primers as follows: *nifH* forward primer: AAAGGYGGWATCGGYAARTCCACCAC, *nifH* Reverse primer: TTGTTSGCSGCRTACATSGCCATCAT; *ureC* forward primer: ATHGGYAARGCNGGNAAYCC, *ureC* Reverse primer: GTBSHNCCCCARTCYTCRTG. The primers were designed by Primer Premier 5.0 and synthesized by Sangon Biotech (Shanghai, China).

### 2.10 Statistical analysis

The data were processed and analyzed by SPSS 23.0 and GraphPad Prism 6.02 statistical software. Based on the normality test and homogeneity of variance, the Student-*t*-test was used when two groups of data were compared (Welch’s *t*-test was used when the variance was not homogeneous). One-way ANOVA analysis of variance with the Tukey-Kramer method was used when multiple groups were compared.

## 3 Results

### 3.1 Gut microbiota of different gut segments in the intestine of *Apriona swainsoni*

**FIGURE 1:**
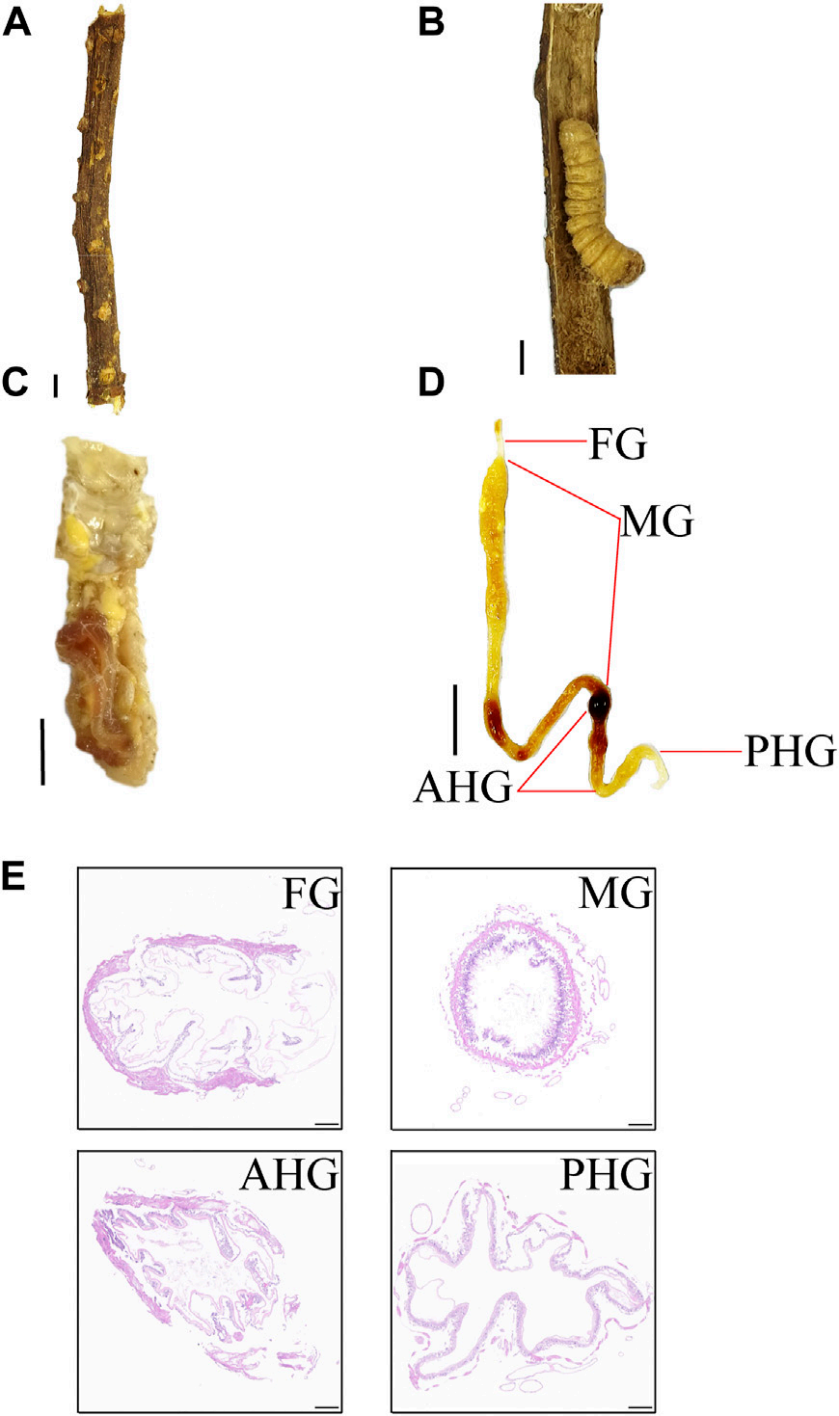
Habitat and gut anatomy and larva of *Apriona swainsoni.* **(A)** *Caesalpinia decapetala* branch. **(B)** An *A. swainsoni* larva in the branch of *Caesalpinia decapetala*. **(C)** Insect body anatomy of *A. swainsoni* larva. **(D)** Depiction of the digestive gut and its four main regions when fully removed and extended. FG, foregut; MG, midgut; AHG, anterior hindgut; PHG, posterior hindgut. **(E)** HE staining of four gut segments. Scale bar, 1 cm (ABCD), 100 µm **(E)**.

Through anatomical analysis, we found that the larva of *A. swainsoni* is about 3 cm long, and its intestinal length is about 11–13 cm long (Figure 1A, B). According to morphological characteristics, the gut can be divided into four segments: foregut (FG), midgut (MG), anterior hindgut (AHG), and posterior hindgut (PHG) (Figures 1C, D). According to HE staining results, the diameter of MG is small, and the intestinal wall of MG is dense and thick. The diameter of PHG is large, and the intestinal wall of PHG is loose and thin. These features of FG and AHG are fallen in between MG and PHG. Thus, the presented results suggested that the morphology of four gut segments is also different, and may function diversely in *A. swainsoni* (Figure 1E).

### 3.2 Multiple omics comparison of gut microbiota of four gut segments

**FIGURE 2:**
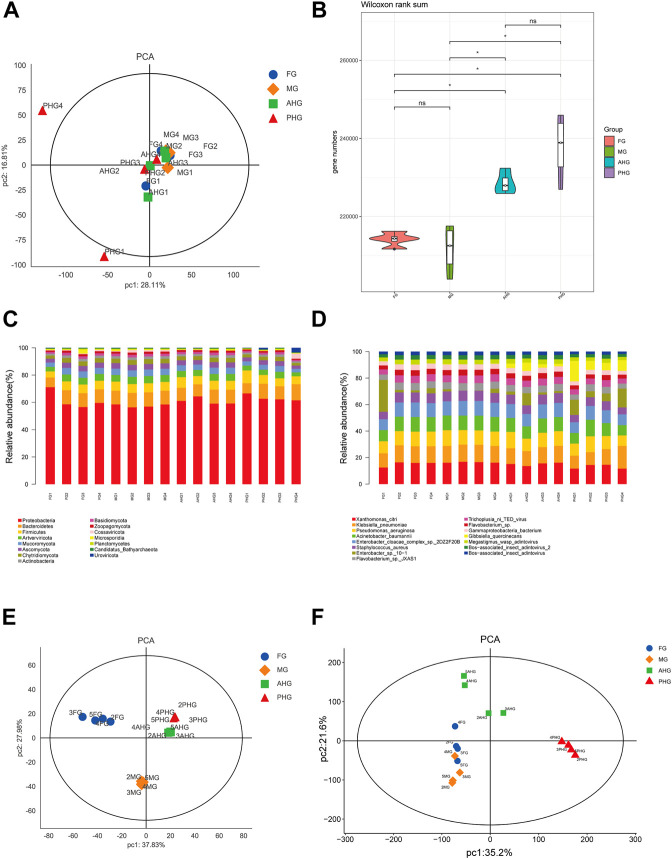
Comparison of multiple meta-omics in four gut segments of *A. swainsoni* larvae. **(A)** Principal component analysis (PCA) of metagenome of four gut segments. **(B)** Violin diagram of metagenome group of four gut segments. Genus numbers of four gut segments were compared by the Wilcoxon method. **(C)** Community barplot histogram at phylum level of four gut segments. Top15 phylum is shown in the barplot. **(D)** Community barplot histogram at the species level of four gut segments. Top15 species are shown in the barplot. **(E)** PCA analysis of metaproteome of four gut segments. **(F)** PCA analysis of metabolome of four gut segments. FG, foregut; MG, midgut; AHG, anterior hindgut; PHG, posterior hindgut.

To understand the effects of different gut segments on larval habitat adaptation, we sequenced and identified the metagenome, metaproteome, and metabolome of four gut segments of the *A. swainsoni* larva. The results of metagenomic principal component analysis (PCA) at the species level suggested that there were no significant differences between samples from different gut segments (Figure 2A), but at the gene number level, there were significant differences between gut segments (*p* < .05), except for foregut and midgut, and foregut and hindgut pairs (Figure 2B). According to taxonomy barplot comparisons, samples from different segments of the larvae differ at both phylum and species levels (Figures 2C, D). Top 15 phylum comparison results showed that there are differences in four gut segments since the eighth rich abundance phylum, i.e., *Basidiomycota*, *Actinobacteria*, *Microsporidia*, *Planctomycetes*, *Cossaviricota*, *Candidatus*, and *Uroviricota.* Top 15 species comparison results showed that there are differences in four gut segments since the fifth rich abundance species especially *Gibbsiella quercinecans*, *Enterobacter cloacae, Flavobacterium* sp.*,* and *Enterobacter* sp. Notably, the results of PCA showed that the metaproteome and metabolome of four segments of *A. swainsoni* larvae were aggregated together and significantly different from other segments, suggesting that the proteome and metabolome of four segments of *A. swainsoni* larvae were significantly different, which may be related to the functional differences of different segments.

To determine crucial microbial organisms in nitrogen fixation and lignocellulose deconstruction across the four gut segments, we performed genome bins by metagenomic data. A total of 22 genome bins were obtained, with 10 bins probably composed of a single bacterial strain with genome contamination ratio lower than 10% and genome completeness larger than 50% (Supplementary Table S1). The taxonomy and the microbial composition of the whole metagenome of these 10 bins were determined through analysis of their bacterial markers (Supplementary Table S1). Notably, all 10 genomes are determined as bacteria. These 10 genome bins were further annotated to verify the existence of functional genes coding for lignocellulose deconstruction enzymes, and nitrogen fixation-related enzymes (Supplementary Table S2). These 10 genome bins were also annotated by the CAZY database to identify the genes coding for glycoside hydrolases enzyme (Supplementary Table S2).

### 3.3 Comparison of metabolite composition in the gut microbiota of four gut segments

**FIGURE 3:**
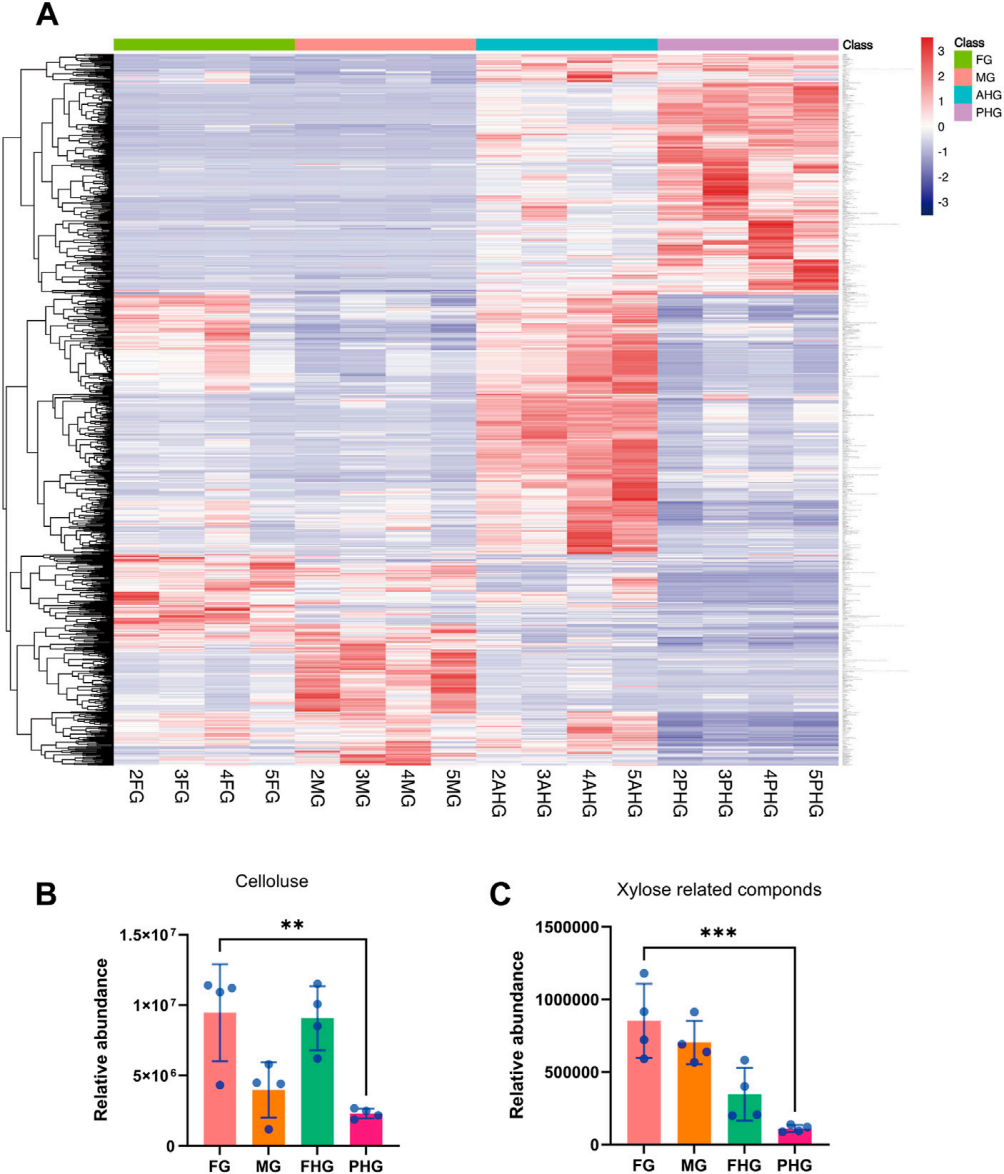
Comparison of featured metabolites in four gut segments of *A. swainsoni* larvae. **(A)** Heatmap of statistical differential metabolites of four gut segments. A total of 1,026 statistically differential metabolites were selected on the basis of the combination of a threshold of variable influence on projection values and *p* values from a two-tailed Student’s *t*-test on the normalized peak areas. **(B)** Comparison of relative abundance of cellulose of four gut segments. **(C)** Comparison of relative abundance of xylose-related compounds of four gut segments. The ** indicates .001 < *p* ≤ .01, and the *** indicates *p* ≤ .001.

We first analyzed the macro metabolome composition of different gut segments and found that a total of 29,130 metabolites were identified in the samples from four gut segments, among which 1,027 metabolites showed significant differences in content in different gut segments (Figure 3A). Further, we found that cellulose content in different gut segments differed significantly (*p* < .01), ranking 31st as the metabolite with significant difference (Supplementary Table S3), and its content was lower in midgut and hindgut, but higher in foregut and foregut (Figure 3B). Interestingly, we also found that hemicellulose content showed a significant gradient of decrease in the four gut segments (*p* < .001), suggesting that metabolism is continuously decomposed with peristalsis of food in the intestinal tract (Figure 3C). Due to the limitations of our metabolomics approach, we were unable to identify differences in lignin, a complex woody component, in different gut segments. Thus, our results indicated that the lignocellulose composition of the *A. swainsoni* larva is distinct in the four gut segments.

### 3.4 Comparison of lignocellulose-related genes and enzymes in four gut segments

**FIGURE 4:**
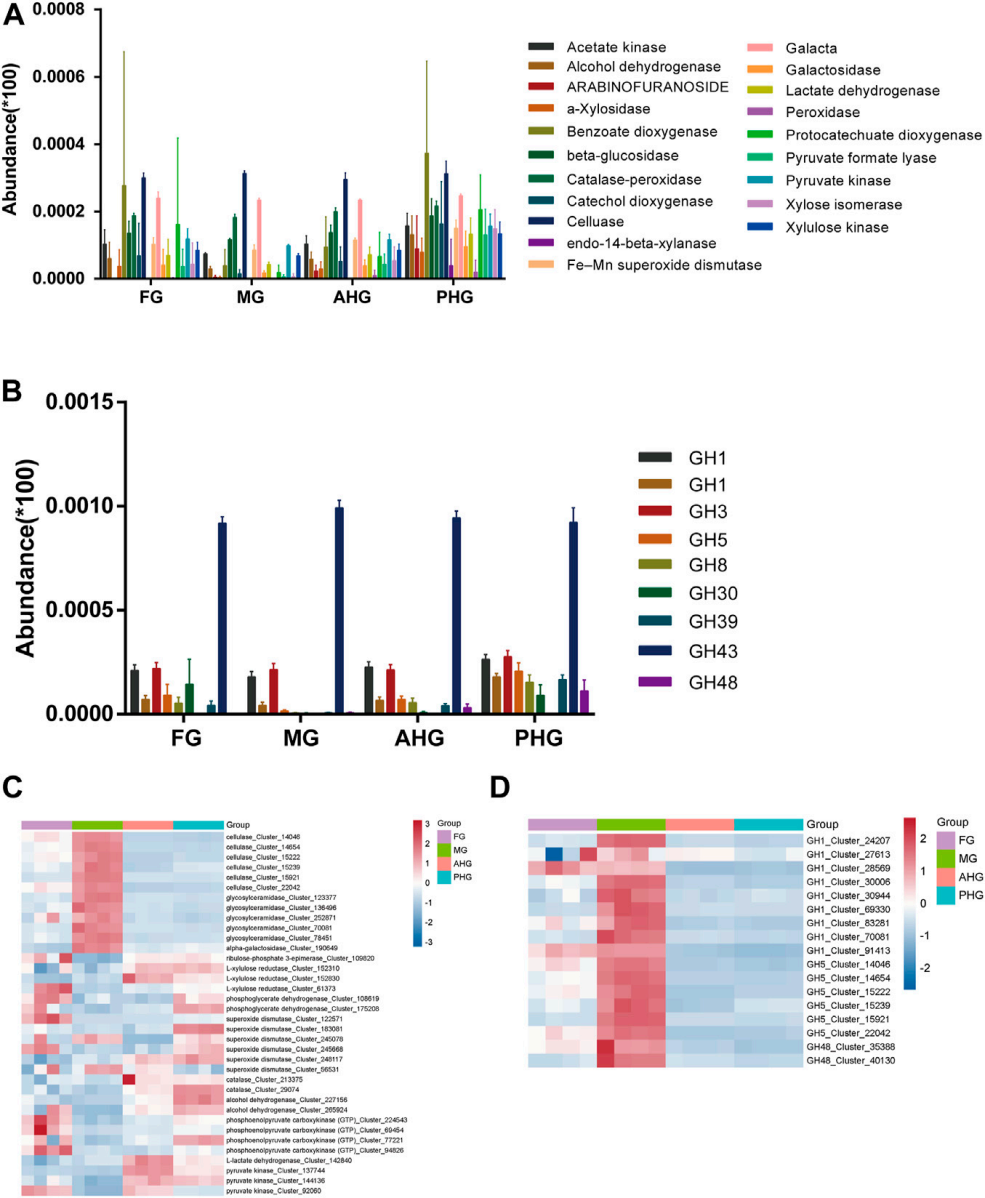
Comparative analysis of lignocellulose degradation-related genes and proteins in four gut segments of *A. swainsoni* larvae. **(A)** Genes coding for different enzymes involved in lignocellulose degradation were detected through the four gut regions of four *A. swainsoni* larvae, with the highest abundance levels detected at the PHG. **(B)** Genes coding for glycoside hydrolases (GH) enzymes were detected through the four gut regions of four *A. swainsoni* larvae, with the highest abundance levels detected at the PHG. **(C,D)**. Metaproteomic survey identified expression of lignocellulose degradation enzymes **(C)**, and 3 GH enzyme **(D)** distributed through the beetle gut with the highest expression level at MG.

According to the results of metabolomics analysis, there were significant differences in lignocellulosic content in four gut segments, so we analyzed the differences between lignocellulosic degradation-related genes and proteases in four gut segments. Metagenomic results (Figure 4A) showed that genes related to lignocellulose metabolism exist in different gut segments. In general, the abundance of related genes was high in the hindgut. According to CAZY annotation analysis (Figure 4B), we found that glucoside hydrolase was also widely distributed in different gut segments. Overall, the abundance of GH genes in the PHG segments was greater than in others, but the abundance of GH1 and GH5 was similar in different gut segments. Interestingly, according to functional Metaproteomic analysis, the expression of cellulase responsible for cellulose metabolism was higher in the midgut segment than in other segments, which may lead to the phenomenon of low cellulose content in the midgut segment found in the metabolomics results. Furthermore, analysis of the expression levels of enzymes related to hemicellulose and lignin metabolism showed that these enzymes showed high expression at the poles of different gut segments and low expression in the middle, which may lead to the gradient decrease of hemicellulose content found in the metabolomics results. It is worth noting that our analysis found that the expression level of GH enzymes identified in the Metaproteomic was significantly higher in the midgut than in other gut segments, similar to the distribution characteristics of cellulase, suggesting that the midgut is the main place for the metabolism of cellulose and related metabolites and sugars. Interestingly, according to the annotation of bin genomes, the genes coding for cellulase are prevalent in bins of four gut regions but are the most abundant in PHG which might explain the lowest abundance of cellulose in PHG (Figure 2C). Therefore, these multi-omics findings suggest that the gut microbiome of *A. swainsoni* in four gut segments may cooperate to attribute to lignocellulose deconstruction.

### 3.5 Comparison of nitrogen-fixing genes in four gut segments

**FIGURE 5:**
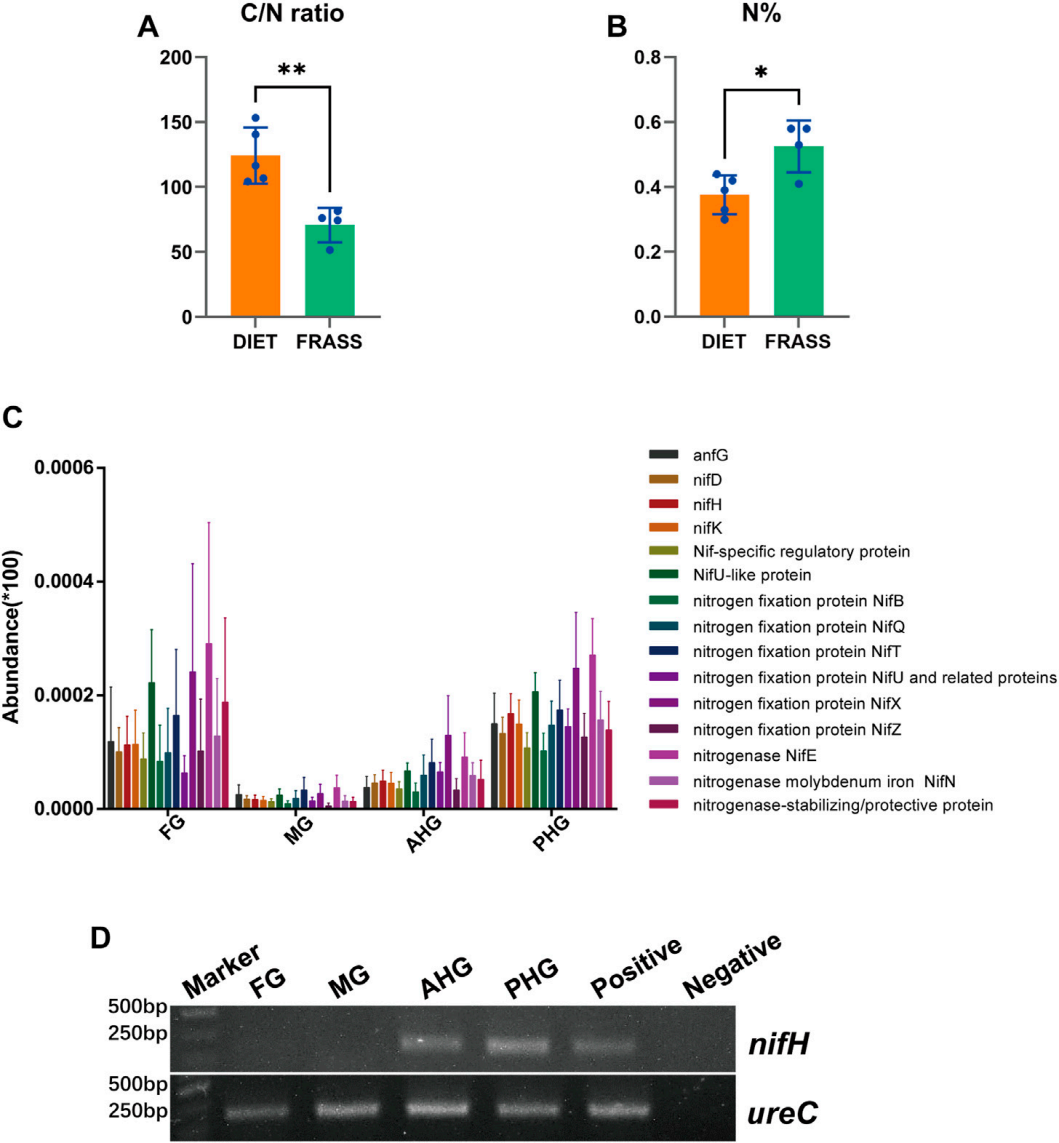
Comparative analysis of nitrogen fixation-related genes and their expression in four gut segments of *A. swainsoni* larvae. **(A,B)** Comparison of carbon/nitrogen (C/N) ratio **(A)** and nitrogen concentrations **(B)** in wood and gut tissue of *A. swainsoni* larvae. **(C)** Genes coding for enzymes involved in nitrogen fixation were detected through the four gut regions of four *A. swainsoni* larvae, with higher abundance levels detected at FG and PHG. **(D)** RT-PCR of key nitrogen fixing gene *nifH. UreC* gene expression was also detected. The * indicates *p* ≤ .05, and the ** indicates .001 < *p* ≤ .01.

Here we aim to determine whether the different gut segments of *A. swainsoni* larvae are involved in nitrogen fixation. We first found that the C/N ratio of woody food of *A. swainsoni* larvae was significantly lower than that of frass. The C composition results were consistent with C/N ratio results (Figure 5A, B). Through analyzing the abundance of nitrogen-fixing genes in metagenomes of four gut segments (Figure 5C), we found that in general, nitrogen-fixing genes showed high abundance at the poles (FG and PHG) and low abundance in the middle of four gut segments (MG and AHG), similar to the distribution of hemicellulose degradation genes. Consistently, although nitrogen fixing genes are founded in all bins of four gut regions, the bins in PHG carry the most amount of nitrogen fixing genes. Unfortunately, due to the limitations of the metaproteomic technique, we were unable to identify nitrogenase expression. As a substitute, we analyzed the expression of the nitrogen-fixing core gene *nifH* (Zehr et al., 2003) in four gut segments (Figure 5D), and surprisingly found that *nifH* only expresses in AHG and PHG segments. In conclusion, metagenomic and gene expression results showed that nitrogen fixation genes mainly existed and expressed in two hindgut segments, suggesting a featured division of nitrogen fixation in gut segments of *A. swainsoni* larvae.

## 4 Discussion

Here, we first found that the morphology of four spatially separated gut segments were different, and may function diversely in *A. swainsoni* (Figure 1)*.* Then, our presented results indicate the metagenomes of four gut segments of *A. swainsoni* larvae are not significantly different in general (Figure 2A). However, four gut segments have different combinations of dominant bacteria and genes (Figures 2B, C, D). The proteome and metabolome of four segments of *A. swainsoni* larvae were significantly different, which may be related to the functional differences of different segments (Figures 2E, F). Furthermore, the metabolome results showed that significant gradient differences exist in the contents of cellulose and xylose in different segments of *A. swainsoni* (Figure 3), and the expression of corresponding metabolic proteins was the highest in the midgut, suggesting the metabolic characteristics of these lignin components in the gut of *A. swainsoni* larvae (Figure 4). Finally, the C/N ratio of woody food of *A. swainsoni* was significantly lower than that of frass (Figure 5A) and metagenomic and gene expression results showed that nitrogen fixation genes mainly existed in foregut and two hindgut segments, and expressed in two hindgut segments (Figure 5C). The results suggested that *A. swainsoni* larvae possessed a featured division of nitrogen fixation in four gut segments. Therefore, our presented results indicated that the gut microbiota of four gut segments may contribute to lignocellulose deconstruction and nitrogen fixation of the larva of *A. swainsoni*.

Gut microbiomes are known to reinforce the niche fitness of many insects, especially wood-feeding insects such as termites and cerambycid (Wong and Rawls, 2012; Karimi et al., 2018). However, host’s ability to establish beneficial microbial partners requires more than the acquisition of the microbes. Hosts must provide various habitats developed through co-evolutionary processes to sustain and promote the microbial metabolic functions of their arthropods and colony composition. In different arthropods and ruminants, host-microbe associations play an important role in lignocellulose energy extraction (Wong and Rawls, 2012; Karimi et al., 2018). However, most have either analyzed the entire gut (or pooled samples in the case of small arthropods) or considered only larger compartments of the gut (as in the case of some termites and beetles) (Bird et al., 2019). Other digestive tract regions may play a role in biomass transformation and nutrient acquisition but are usually overlooked. Here, our study examines the interaction between gut anatomy and the distribution of microbial processes for lignocellulosic biomass transformation through the four major gut regions in the wood-feeding larvae of *A. swainsoni*. There is considerable variation in the morphology and other physicochemical properties of the digestive tracts of *A. swainsoni* larvae, factors that are likely to have a significant impact on the composition of the gut microbial community. Based on our results, the digestive tract anatomy of *A. swainsoni* has shaped conditions favoring microbial metabolic processes that enable subsistence on a recalcitrant diet. For the effective use of woody biomass as only energy and source, deliciate metabolism must occur for lignocellulose deconstruction and nitrogen fixation, with spatial compartmentalization reinforcing their relative efficiency while allowing essential coupling between these processes. We propose that the anatomical properties of *A. swainsoni* enable this through the promotion of lignocellulose deconstruction and nitrogen fixation can co-occur and feed fermentative processes. Our presented results may benefit the finding of potential biocontrol or pesticide action targets for future Cerambycid control research and application.

Recently, Ceja-Navarro and colleagues found that the wood-feeding passalid beetles (*Odontotaenius disjunctus*) sharing similar living niches of *A. swainsoni* harbor highly complex gut microbiota sequentially across compartments which functions the degradation of lignocellulose and nitrogen fixation (Ceja-Navarro et al., 2019). Their results indicate that the wood-feeding lifestyle of the beetles requires both the formation of specific habitats within the host and the acquisition of microorganisms to promote unique and efficient microbial metabolic functions. Here, we found that in *A. swainsoni* larvae, three GH proteins (GH1, GH5, and GH48), as well as cellulase, expressed mainly at MG segments, which are similar to passalid beetles. Thus, these findings suggest that both two wood-feeding insects may share a similar lignocellulose deconstruction pattern for survival in the wood. Interestingly, our presented results indicated that nitrogen fixation genes mainly existed in the foregut and two hindgut segments, and were expressed in two hindgut segments of *A. swainsoni* larvae. However, Nitrogenase expression in *O. disjunctus* gut was highest in the AHG segment (Ceja-Navarro et al., 2019). These findings may indicate that the two insects evolve two distinct gut microbiomes to fix the nitrogen in the xylem of trees. Therefore, our findings suggest that wood-feeding insects may evolve distinct or similar lignocellulose deconstruction and nitrogen fixation with the gut microbiota to live in the xylem of trees.

In conclusion, our presented results provide evidence that the larvae of *A. swainsoni* can adapt to the relatively harsh niche conditions through the highly organized gut microbiome of four gut segments, and play a major role in nutritional absorption. Further research about gut microbiota in gut parts of adults and pupae of *A. swainsoni* is worth performing in the future to shed light on the whole life cycle of physical features and functions of gut microbiota in *A. swainsoni* and other Longhorn beetles.

## Data Availability

The datasets presented in this study can be found in online repositories. The names of the repository/repositories and accession number(s) can be found in the article/Supplementary Material.
